# Solvent-Free Fabrication of Biphasic Lipid-Based Microparticles with Tunable Structure

**DOI:** 10.3390/pharmaceutics14010054

**Published:** 2021-12-27

**Authors:** Serena Bertoni, Beatrice Albertini, Joanna Ronowicz-Pilarczyk, Natalia Calonghi, Nadia Passerini

**Affiliations:** 1 Department of Pharmacy and BioTechnology, Pharm Tech Lab, University of Bologna, Via S. Donato 19/2, 40127 Bologna, Italy; serena.bertoni4@unibo.it (S.B.); nadia.passerini@unibo.it (N.P.); 2 Department of Inorganic and Analytical Chemistry, Faculty of Pharmacy, Collegium Medicum in Bydgoszcz, Nicolaus Copernicus University in Toruń, Jurasza 2, 85-089 Bydgoszcz, Poland; joanna.ronowicz@gmail.com; 3 Department of Pharmacy and BioTechnology, Cancer Metabolism Lab, University of Bologna, Via Irnerio 48, 40126 Bologna, Italy; natalia.calonghi@unibo.it

**Keywords:** spray chilling, spray cooling, melt emulsification, core-shell, microemulsion, interfacial tension, PEG, long chain glycerides, Raman mapping, oral drug delivery

## Abstract

Lipid-based biphasic microparticles are generally produced by long and complex techniques based on double emulsions. In this study, spray congealing was used as a solvent-free fabrication method with improved processability to transform water-in-oil non-aqueous emulsions into spherical solid lipid-based particles with a biphasic structure (b-MPs). Emulsions were prepared by melt emulsification using different compositions of lipids (Dynasan^®^118 and Compritol^®^888 ATO), surfactants (Cetylstearyl alcohol and Span^®^60) and hydrophilic carriers (PEGs, Gelucire^®^48/16 and Poloxamer 188). First, pseudo-ternary phase diagrams were constructed to identify the area corresponding to each emulsion type (coarse emulsion or microemulsion). The hydrophobicity of the lipid mostly affected the interfacial tension, and thus the microstructure of the emulsion. Emulsions were then processed by spray congealing and the obtained b-MPs were characterized in terms of thermal and chemical properties (by DSC and FT-IR), external and internal morphology (by SEM, CLSM and Raman mapping). Solid free-flowing spherical particles (main size range 200–355 µm) with different architectures were successfully produced: microemulsions led to the formation of particles with a homogeneous internal structure, while coarse emulsions generated “multicores-shell” particles consisting of variable size hydrophilic cores evenly distributed within the crystalline lipid phase. Depending on their composition and structure, b-MPs could achieve various release profiles, representing a more versatile system than microparticles based on a single lipid phase. The formulation and technological strategy proposed, provides a feasible and cost-effective way of fabricating b-MPs with tunable internal structure and release behavior.

## 1. Introduction

Solid lipid microparticles (SLMs) are micro-scale drug delivery systems possessing a matrix made of high melting points lipids, such as fatty acids, glycerides, fatty alcohols, or solid waxes. Lipid excipients present important advantages, such as ease of large-scale production, low cost, ease of manufacturing and they are completely tolerated by the body due to their non-cytotoxic and biodegradable nature [1]. SLMs are mostly employed to control and prolong the release of drugs [2].

Recently, the increasing number of drug molecules with challenging delivery issues (e.g., dual delivery, controlled release with minimum burst effect, delivery of labile compounds) has amplified the demand for new carrier systems able to effectively achieve the desired release behavior. Therefore, the research has progressed toward the production of lipid-based systems with more complex structures and architectures [3]. Hence, instead of a simple lipid matrix, hybrid or multicompartment particles composed of lipids coupled with a different type of material have been developed in order to combine several properties in a single formulation [4,5,6,7]. The combination of a chemically different core material enclosed within a lipid shell has potential for tunable release performances, excellent drug protection and co-delivery of drugs, properties that are not achievable separately by the lipid and the core material alone [8]. For example, hybrid particles consisting of polymers combined with lipid-based materials have been developed to optimize the release profile of the loaded API [9,10].

The production methods of biphasic lipid-based microparticles for drug delivery applications are usually based on the formation of double emulsions. Double emulsions are prepared via two steps: in the first step water droplets are dispersed in the oil phase to form a primary water-in-oil (W/O) emulsion, and then the primary emulsion is dispersed into a secondary aqueous solution (W) to form the double (W/O/W) emulsion. This process is quite complex and suffers from poor batch-to-batch reproducibility, low encapsulation efficiency and insufficient method scalability [11]. Moreover, the presence of organic solvents seriously limits the biocompatibility of the resulting particles [12]. One possible way to overcome this problem is by using a melted material rather than a solution of the lipid in an organic solvent. In this case, the low-melting material is heated above its melting point during emulsion preparation and then undergoes a liquid-to-solid transition by lowering the temperature [13]. This method, called melt emulsification or melt dispersion, has been successfully employed for the encapsulation of small molecules drugs [14,15,16,17], or peptides [18,19] into SLMs. Although the melt emulsification method excludes the use of organic solvents, making the process environmentally friendly, one or more additional step(s), such as filtration, centrifugation, or lyophilization is needed in order to obtain water-free particles [20]. Moreover, melt emulsification processes employing double (W/O/W) emulsions are generally associated with drug loss during emulsification and poor drug loading, especially for water-soluble drugs. Therefore, a novel approach for the “green” preparation of biocompatible multicompartment microparticles with an easy, robust and reproducible production process is highly desired.

Herein, we explored an innovative approach for the preparation of multicompartment lipid-based microparticles by spray congealing technology. Spray congealing is a manufacturing method of SLMs based on the atomization of a molten fluid and its solidification upon cooling [21,22,23]. The key benefits of this technology are the absence of solvent, either aqueous or organic, and the possibility to fabricate spherical particles without the need for other downstream processes (e.g., drying or spheronization) [22]. The low-melting materials commonly employed include lipid-based excipients derived from vegetable glycerides, fatty acids, or waxes, as well as hydrophilic materials, such as polyethylene glycols, poloxamers and high HLB Gelucires^®^. To date, spray congealing technology has been employed exclusively using one-phase material as a carrier and the resulting SLMs consisted in “matrix” systems, either hydrophobic or hydrophilic-based, with the loaded compound evenly distributed (molecularly dispersed or not) within the particle volume [24,25,26]. In this case, the release behavior is mainly affected by the chemical nature of the drug and excipient, the drug solid state and concentration, and possible drug-carrier interaction [27].

Therefore, the possibility to use spray congealing to transform a melt emulsion, specifically a single non-aqueous W/O emulsion, into spherical solid particles with a biphasic structure (b-MPs) was here investigated for the first time. In particular, single emulsions were first developed using commercially available and pharmaceutically approved components commonly used for spray congealing and their formation was monitored by the corresponding pseudo-ternary phase diagram. The oil phase was composed of lipophilic materials (e.g., long chain glycerides), having melting temperatures (T_m_) above room temperatures, while non aqueous hydrophilic materials either liquid (e.g., low molecular weight-polyethylene glycol (PEG) or solid (e.g., high molecular weight-PEG, Gelucire^®^48/16, Poloxamer 188) were used as the inner phase of the emulsion. Different low HLB surfactants (Cetylstearyl alcohol and Span^®^60) and a high HLB co-surfactant (Cremophor^®^EL) were also investigated to define the region of emulsion formation according to the relative amount of all components. Rhodamine (Rho) was added to the hydrophilic phase as a fluorescent probing dye. Selected emulsions were then atomized to obtain the b-MPs and their particle size, thermal and chemical properties, external morphology and internal structure were investigated. Complementary techniques (cross-sectional SEM images, confocal laser scanning microscopy (CLSM) and Raman mapping) were employed to provide a complete picture of the MPs internal structure and to study the spatial distribution of the two phases. Finally, in vitro release behavior of b-MPs was investigated.

## 2. Materials and Methods

### 2.1. Materials

Dynasan 118 (tristearin) was obtained from IOI OLEO GmbH (Hamburg, Germany). Compritol 888 ATO and Gelucire^®^48/16 were kindly supplied from Gattefossè (Milan, Italy). Kolliphor^®^ P188 (Poloxamer 188) and Kolliphor^®^ EL, formerly known as Cremophor^®^ EL (Polyoxyl 35 Hydrogenated Castor Oil), were a gift of BASF. Cetylstearyl alcohol, polyethylene glycol (PEG) 400, PEG 4000 and rhodamine B (Rho) were purchased from Sigma Aldrich (Steinheim, Germany). All other chemicals were of analytical grade. The properties of the hydrophobic and hydrophilic compounds are reported in Table 1.

### 2.2. Study of the Non-Aqueous Emulsions

#### 2.2.1. Preparation of the Emulsions

Emulsions were prepared varying the amount of hydrophobic phase, hydrophilic carrier and co-surfactant. In particular, 3:1 or 6:1 lipid:hydrophobic surfactant ratios were used, based on preliminary experiments. As for Dynasan-based emulsions, only a 3:1 ratio of lipid and lipophilic surfactant was used as the lower amount of surfactant did not allow the emulsion formation and the hydrophilic and hydrophobic phases were completely separated, while for Compritol-based emulsion both ratios were tried. Considering the different materials used as excipients, the emulsions were labeled according to the component types. Specifically, the first letter corresponded to the lipid (Dynasan, D and Compritol, C), the second letter to the main surfactant (Cetylstearyl alcohol, C and Span 60, S), the third and fourth letters to the hydrophilic carrier (PEG 400, Pl; PEG 4000, Ps; Gelucire, Ge and Poloxamer, Po), followed by a number identifying a specific composition of the hydrophilic carrier and co-surfactant. The composition of the prepared Dynasan-based and Compritol-based emulsions are reported in Table 2 and Table 3, respectively.

Batches of 5 g of emulsions were prepared. The hydrophobic phase (lipid and hydrophobic surfactant) was heated at a temperature of 5 °C above the melting point of the lipid, using temperatures in the range of 70–80 °C. The hydrophilic phase was heated at the same temperature (70 °C) if liquid, or melted if solid, added to the lipid phase and gently mixed (250 rpm) via a magnetic bar keeping the temperatures at 70 °C using a hot stirring plate for at least 1 min. For Dynasan-based emulsions, cloudy emulsions were obtained. Using Compritol as lipid and PEG 400 as a hydrophilic component, two different types of emulsions were observed: cloudy and transparent. Therefore, pseudo-ternary phase diagrams were constructed for these formulations.

#### 2.2.2. Ternary Phase Diagram of Compritol-PEG 400 Based Emulsions

Pseudo-ternary phase diagrams were constructed according to the work of Constantinides and Scalar [28]. In this phase diagram, the hydrophobic phase (mixture of the lipid and low HLB surfactant) is shown at the top of the phase diagram, while the high HLB surfactant and the hydrophilic carrier are shown on the bottom left and right corner, respectively. The weight ratio of the lipid to the hydrophobic surfactant was kept constant (3:1 or 6:1), with varying amounts of the hydrophilic phase. As the hydrophobic phase amount in the emulsion was always equal to or higher than 50% *w/w*, only the upper part of the phase diagram was considered.

For the construction of the phase diagram, the prepared emulsions (Table 3) were added to the diagram. To determine boundaries between regions corresponding to different emulsion classes, additional emulsion samples (3 g) were prepared and observed. To have a clearer visualization of the emulsion structure, rhodamine (Rho) was added to the hydrophilic phase as a water soluble dye (pink color). The classification of these additional samples as transparent or cloudy allowed us to determine the areas of the phase diagram that corresponded to different emulsion types.

#### 2.2.3. Hot Stage Microscopy (HSM) Analysis

Melt emulsions were observed under optical microscope using a Nikon Eclipse E400. A hot stage apparatus (Mettler-Toledo S.p.A., Novate Milanese, Italy) was used to keep the sample at 70 °C during analysis. The micrographs of emulsions were recorded using a Nikon Digital Net Camera DN100 connected to the microscope at a magnification of 10×.

#### 2.2.4. Hot Stage-Polarized Light Microscopy (HS-PLM) Analysis

Thin films of emulsions were observed under polarized light with a Nikon Eclipse E400 optical microscope connected to a Nikon Digital Net Camera DN100 for image acquisition. For sample preparation, one drop of the emulsion was added to a heated glass slide, the coverslip was applied. The films were then heated by the hot stage apparatus (Mettler-Toledo S.p.A., Novate Milanese, Italy) and images were taken at 70 °C. Then, the samples were let solidify and images were taken at 25 °C.

#### 2.2.5. Interfacial Tension Measurements

The interfacial tension (IRT) between the hydrophilic and lipophilic phases was measured by a tensiometer (Kruss tensiometer K8600, Hamburg, Germany) with the Du Noüy ring method, similarly to previously reported studies [29]. First, the accuracy of the platinum-iridium ring was validated by determining the surface tension of water (72 mN/m). For these measurements, 2 mL of the oil phase was transferred in a glass vessel which was thermostated at 70 °C by a water bath. To avoid mixing of the oil and water phase, the hydrophilic phase (PEG 400) was carefully pipetted on top of the oil phase (2 mL). Measurements were repeated five times for each sample.

### 2.3. Biphasic MPs Production by Spray Congealing

**Figure 1:**
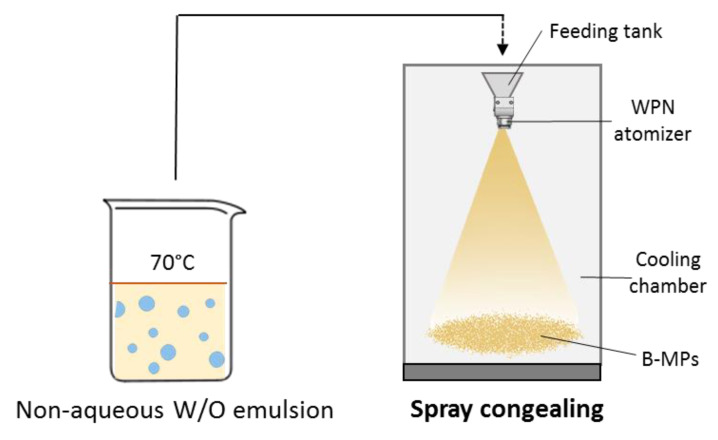
Schematic Illustration of the fabrication process of the b-MPs: Transformation of the non-aqueous emulsion into solid particles by spray congealing technology.

Spray congealing was employed to obtain solid b-MPs, as schematized in Figure 1. The spray congealing apparatus comprises a feeding tank, an atomizer and a cooling chamber. An external-mix two-fluid atomizer, called Wide Pneumatic Nozzle (WPN), was used [30]. Emulsions were prepared according to Section 2.2 and stirred keeping the temperatures at 70 °C. Then, the hot emulsions were introduced into the feeding tank of the spray congealing apparatus. All emulsions (Table 2 and Table 3) were atomized at 1.5 bar air pressure and nozzle temperature of 70 °C. The atomized molten droplets solidified in the cooling chamber at room temperature (25 °C), allowing the formation of solid particles. MPs were collected from the bottom of the cooling chamber and stored in polyethylene closed bottles at 25 °C. The percentage yield was calculated using the following Equation:

Yield=(Amount of recovered MPs (g))/(Amount of emulsion (g)) ×100

For the preparation of fluorescent Rho-loaded b-MPs, Rho at a concentration of 0.2% *w/w* was solubilized into the hydrophilic phase prior to emulsion formation. b-MPs were prepared following the production process described above.

In addition, formulations of lipid microparticles (SLMs) constituted of a one-phase lipid matrix were prepared using both Compritol^®^888 ATO and Dynasan 118 as lipids. Rho was added at 0.2% *w/w* into the molten carrier. The process parameters were the same as those used for the production of b-MPs.

### 2.4. Characterization of b-MPs

#### 2.4.1. Differential Scanning Calorimetry (DSC)

DSC analysis was performed using a Perkin Elmer DSC 6 (Perkin Elmer, Beaconsfield, UK) with nitrogen as purge gas (20 mL/min). The instrument was calibrated with indium and lead for temperature, and with indium for the measurements of the enthalpy. Samples weighing 5–8 mg of b-MPs were placed in aluminum pans and heated from 30 to 90 °C at a scanning rate of 10 °C/min. As a reference, raw excipients were submitted to two consecutive heating processes. This enabled us to determine the thermal profiles of both the thermodynamically stable forms (first heating) and of the form solidified by cooling from the melt (second heating).

#### 2.4.2. Fourier Transform-Infrared Spectra (FT-IR)

Studies of infrared spectra of raw materials and b-MPs were conducted with an IR spectrophotometer (Jasco FT-IR A-200, Milan, Italy) using the KBr disc method. The samples were mixed with KBr and compressed into a tablet (10 mm in diameter and 1 mm in thickness) using a manual hydraulic tablet presser (Perkin Elmer, Norwalk, CT, USA) at 4000 kg/cm for 4–6 min.

#### 2.4.3. Particle size

Size distribution of b-MPs was evaluated by sieve analysis using a vibrating shaker (Octagon Digital, Endecotts, London, UK) and a set of six sieves ranging from 50 to 500 μm (Scientific Instrument, Milan, Italy).

#### 2.4.4. Scanning Electron Microscopy (SEM)

The shape and surface morphology of b-MPs were assessed on freshly prepared particles by means of SEM. Moreover, in order to observe the internal morphology of the particles, b-MPs with diameters ~500 µm were carefully cut with a type 21 scalpel. Both samples were fixed on the sample holder with double-sided adhesive tape. SEM images were obtained using a Zeiss Scanning Electron Microscope Model EVO 50 EP. The samples were observed without coating in EP mode with a chamber pressure of 90 Pa and an acceleration voltage of 10 kV.

#### 2.4.5. Confocal Laser Scanning Microscopy (CLSM)

The internal structure of b-MPs was investigated using fluorescent Rho-labeled b-MPs. The b-MPs were dispersed in paraffin oil and placed onto a glass slide and multiple images were acquired by using sequential laser excitations at 568 nm to reduce spectral bleed-through artifacts. The images were collected by using a Nikon C1s confocal laser-scanning microscope (Nikon, Minato, Tokyo, Japan) equipped with a Nikon PlanApo 60X, 1.4-NA oil immersion lens.

#### 2.4.6. Raman Mapping

Raman imaging was used for the evaluation of the spatial distribution of hydrophilic and lipophilic phases in the microparticles. A WITec Alpha 300 Raman spectrometer was used to generate Raman spectra of the samples in the range of 10–3600 cm^−1^, with a spectral resolution of 3 cm^−1^. Raman spectrometer was connected with a confocal microscope, equipped with TrueSurface attachment and EMCCD detector for ultra-fast and sensitive imaging. The samples were irradiated with a focused laser beam at a power of 15 mW. The laser was emitting at the wavelength of 532 nm. The b-MPs were mapped with a spatial resolution of 5 µm. The measuring step was set to 1.67 µm, the accumulation time of a single spectrum was 0.5 s. Before Raman mapping, Raman spectra for all used raw materials were recorded in order to identify key marker bands for the identification of excipients in the investigated samples. No special sample preparation was needed and the applied Raman mapping technique was non-destructive for the samples. The collected Raman mapping data was analyzed by means of the Project Four 4.1 Plus software. The analysis based on the calculation of the integral value (area under the band) for the selected bands as well as chemometric analysis (k-means cluster analysis) were performed in order to describe a distribution of hydrophilic and lipophilic phase in the investigated- MPs.

### 2.5. Rho Content and In Vitro Release Studies from Biphasic MPs

Rho loading into b-MPs was determined by adding 5 mg of b-MPs in 5 mL of distilled water, heated to 70 °C to melt the solid carrier and centrifuged. The supernatant was diluted with the same solvent and the amount of Rho was determined by fluorescence measurements. The emission intensity at 574.0 nm was recorded using a Jasco FP-750 spectrofluorometer (Tokyo, Japan) using an excitation wavelength of 553.5 nm. The excitation and emission fluorescence spectra of Rho are reported in Figure S1, Supplementary Materials. The Rho calibration curve was previously determined and was linear in the concentration range 0.02–0.60 µg/mL (R2 = 0.9988) (Figure S2).

In vitro release profiles were assessed using the Apparatus 2 (paddle apparatus) of the Eur. Ph. (DT 800 Erweka GmbH, Heusenstamm, Germany) rotating at 50 rpm. An exactly weighted amount of about 100 mg of b-MPs was added to 500 mL of distilled water at 37 °C in order to have about 0.2 μg/mL of Rho. As drug release is influenced by particle size, the same size fraction (250–355 µm) was used for all samples. During the dissolution tests, 2 mL of aliquots were withdrawn at predetermined time intervals (1, 5, 10, 15, 30, 45, 60, 90 and 120 min) from the dissolution medium using an 8 μm filter to avoid the removal of solid b-MPs and replaced with fresh medium. The amount of Rho released was determined by fluorescence measurements following the same analytical method used for Rho content determination.

## 3. Results and Discussion

### 3.1. Stability and Structure of the Emulsions

**Figure 2:**
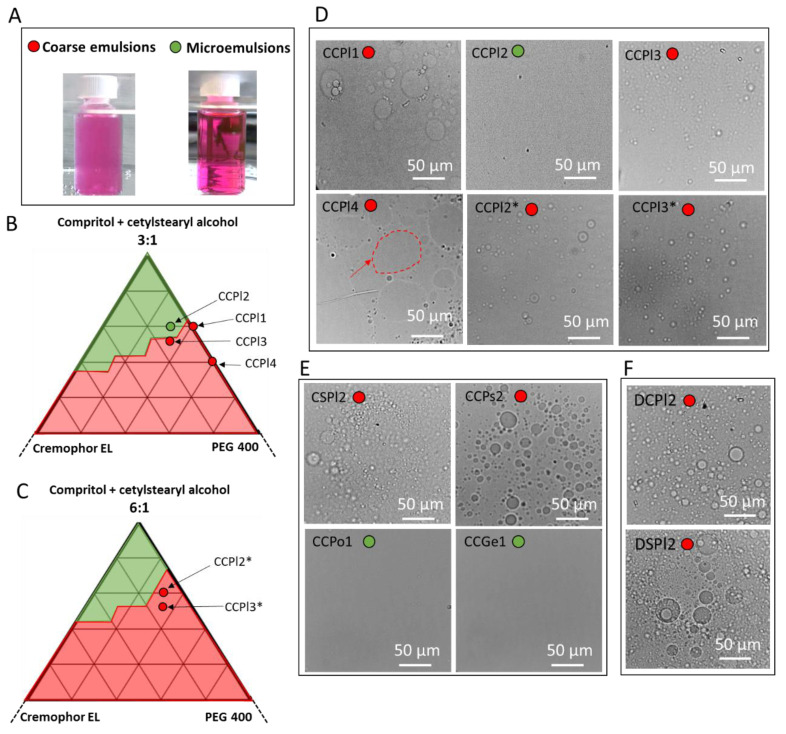
Appearance of the non-aqueous emulsions observed at 70 °C: emulsions (cloudy) and microemulsions (transparent). The hydrophilic phase is loaded with a probing pink dye (Rho) **(A).** Pseudo-ternary phase diagrams of emulsions with Compritol 888 ATO:cetylstearyl alcohol at 3:1 ratio (**B**) and 6:1 ratio (**C**). Optical microstructure of emulsions with Compritol 888 ATO:cetylstearyl alcohol (**D**). Effect of a different surfactant (CSPl2) and different hydrophilic carriers (CCPs2, CCPo1 and CCGe1) on the microstructure of Compritol 888 ATO-based emulsions (**E**). Optical microstructure of Dynasan 118-based (DCPl2 and DSPl2) emulsions (**F**).

Insights were first provided into the effect of the type of lipid on the emulsion formation. According to their appearance, the non-aqueous W/O emulsions obtained were observed to fall under two categories, as shown in Figure 2A: cloudy or transparent systems.

Most formulations, including all Dynasan 118-based emulsions (Table 2) and the larger part of the Compritol-based emulsions (Table 3) were cloudy. Nevertheless, some formulations containing Compritol:Cetylstearyl alcohol 3:1 as hydrophobic phase and PEG 400 as hydrophilic phase were completely transparent. Therefore, additional emulsion samples were prepared and observed in order to determine the boundaries of the areas that corresponded to the two emulsion types shown in Figure 2A. The partial pseudo-ternary phase diagram of emulsions at 3:1 and 6:1 weight ratios of Compritol:Cetylstearyl alcohol are summarized in Figure 2B and Figure 2C, respectively. The upper-left area of the diagram corresponded to clear transparent emulsions (green area in Figure 2B, C). This type of clear stable emulsion was hypothesized to consist of a microemulsion, which is an isotropic and thermodynamically stable liquid biphasic system. The examination under optical microscopy of these emulsions showed the presence of uniform systems (CCPl2 in Figure 2D), consistently with the microemulsion structure, characterized by nano-sized droplets. Additional experiments showed that the emulsions classified as transparent formed spontaneously when their components were mixed, even by changing the order of addition of the components and without the application of high energy mixing, supporting the hypothesis of microemulsion formation.

The microemulsion area of Figure 2B was more extended compared to the one produced by using the 6:1 ratio (Figure 2C). Thus, the amount of cetylstearyl alcohol influenced the emulsion structure acting as a stabilizer of the interface of the inner droplet, with increasing amounts favoring the formation of microemulsions.

Close to the green area, a wide red area indicated emulsions with a cloudy appearance. The corresponding micrographs were characterized by spherical inner phase droplets with non-uniform sizes ranging from 1–10 µm to about 40 µm (e.g., CCPl1 in Figure 2D). By further increasing the amount of PEG 400 (e.g., CCPl4 in Figure 2D), the emulsion microstructure was characterized by large (10–60 µm) droplets with the irregular shape of the hydrophilic phase (red arrow in Figure 2D). It was observed that cloudy emulsions with very small dispersed droplets (submicron or few micron-sized) were favored by high Cremophor EL amounts (e.g., CCPl3).

The formulation CCPl2 was set as a reference formulation representing the microemulsion system. Further studies were conducted starting from this emulsion by changing its composition (Figure 2E). For example, by changing the hydrophobic surfactant from cetylstearyl alcohol to Span 60 (CSPl2), the emulsion was characterized by multiple spherical droplets. When solid PEG 4000 was used instead of liquid PEG 400 (CCPs2), a system with well-defined spherical droplets was obtained in all areas regardless of the addition of increasing amounts of co-surfactant. Conversely, when solid hydrophilic materials with surface-active properties, such as Poloxamer 188 (CCPo1) and Gelucire 48/16 (CCGe1), were used as the hydrophilic phase, a microemulsion formed even without the addition of Cremophor EL. These results evidenced the significant impact of the hydrophilic phase nature on the properties of the Compritol-based systems.

Finally, the emulsions based on Dynasan 118 as main lipid (DCPl2 and DSPl2) were all cloudy and their microstructures were characterized by spherical droplets of hydrophilic phase with size up to 10–15 µm (Figure 2F).

Overall, two main types of non-aqueous W/O systems were obtained by melt emulsification:

1.  Microemulsions: homogeneous transparent systems formed spontaneously;
2.  Coarse emulsions appear opaque due to the presence of micron- or submicron-sized droplets.

**Figure 3:**
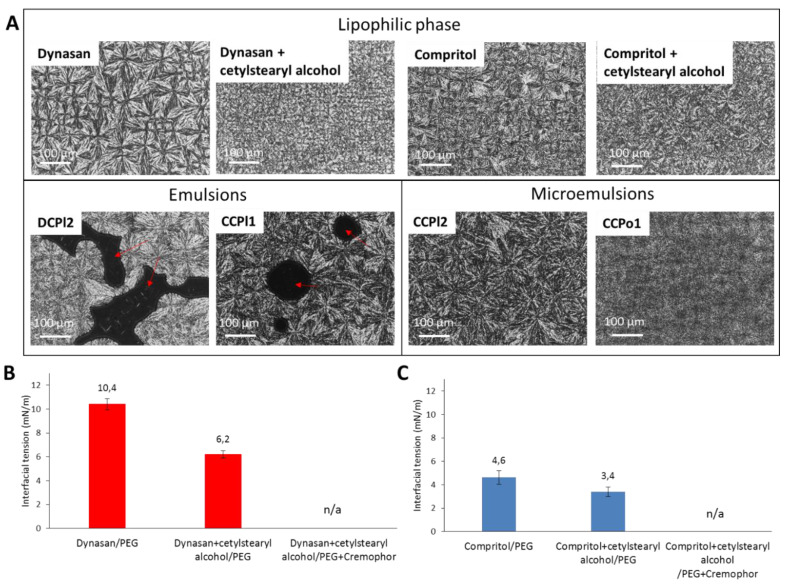
HS-PLM images of lipophilic phase (main lipids without and with cetylstearyl alcohol), examples of Dynasan-based and Compritol-based melt emulsions and Compritol-based melt microemulsions. Dark areas relative to liquid PEG are marked with red arrows. (**A**). Interfacial tension values of Dynasan-based (**B**) and Compritol-based (**C**) biphasic systems.

The non-aqueous W/O systems were also analyzed by HS-PLM and compared with the main lipid alone or with the addition of the hydrophobic emulsifier. Specifically, the morphology of the systems was observed after solidification in a thin layer upon cooling at 25 °C (Figure 3A). Coarse emulsions displayed dark areas under polarized light (red arrows) corresponding to the liquid hydrophilic phase, surrounded by a solid crystalline phase. The crystalline phase of DCPl2 showed a different morphology with respect to that of CCPl1, reflecting the microstructure of the different lipids used. Specifically, the crystalline pattern of Dynasan 118 (tristearin), characterized by spherical well-defined spherulites [31], appeared more ordered than those of Compritol^®^ 888 ATO (mono- (20%), di-(50%) and triesters (30%) of behenic acid), reflecting the molecular heterogeneity of this excipient. The addition of cetylstearyl alcohol to the lipids led in both cases to smaller and less defined spherulites. Conversely to coarse emulsions, microemulsions appeared as homogeneous continuous crystalline matrixes (CCPl2), without any detectable liquid phase. Moreover, a difference in the crystalline morphology of Compritol-based formulations was observed between those containing PEG/Cremophor and those containing Poloxamer (CCPo1), indicating that the hydrophilic carrier distributes within the lipophilic phase affecting the lipid microstructure. Finally, if observed at the liquid state (70 °C), the absence of any specific pattern under polarized light for both emulsion types excludes the presence of liquid crystalline structures (images not shown).

As the lipophilic-hydrophilic phases interface is the most relevant feature governing the chemico-physical behavior of the emulsions, the interfacial tension (IFT) of the investigated systems was measured (Figure 3B, C). As expected, the system Dynasan 118-PEG showed a higher IFT compared to Compritol^®^ 888 ATO-PEG, either with or without hydrophobic surfactant. The addition of cetylstearyl alcohol diminished the IFT and the co-surfactant Cremophor EL further decreased the IFT to values below the limit sensitivity of the instrument. This explains why 5% *w/w* of Cremophor EL was necessary for Dynasan-based systems (e.g., DCPl2) to allow emulsion formation (otherwise not possible) and facilitate the dispersion of the hydrophilic phase in multiple spherical droplets, accordingly to the emulsion structure observed by optical microscopy. Differently, the lower forces involved in the mixing of Compritol and PEG, as indicated by the IFT values always below 5 mN/m, explain the possibility to obtain different types of Compritol-based biphasic systems, including cloudy emulsions as well as microemulsions.

Overall, the results indicate that the lipid was decisive in determining the type of emulsion system formed. In particular, the hydrophobicity of the lipid appeared to be the most important property to affect the interfacial stability of the emulsion droplets and thus the emulsion structure. In fact, irrespective of the hydrophilic phase compositions, a lipid with high hydrophobicity (Dynasan 118) presenting a higher IFT with the hydrophilic phase, led only to cloudy emulsions with visible inner phase droplets. Conversely, a partial glyceride with intermediate polarity (Compritol 888 ATO) could give different types of emulsion according to the formulation composition. In this case, various formulation parameters, such as the lipid to surfactant ratio, the type of surfactant and the amount and composition of hydrophilic phase were of critical importance. Using Compritol 888 ATO as lipid and PEG 400 as a hydrophilic carrier, microemulsions were obtained only with specific hydrophobic phase:PEG 400:Cremophor EL weight ratios.

### 3.2. Production and Characterization of b-MPs

Irrespective of the structure (coarse emulsion or microemulsion) of the starting emulsion, solid spherical particles were obtained. An atomization pressure of 1.5 bar and a nozzle temperature of 70 °C were selected according to the emulsion properties (i.e., low viscosity and melting temperature of the excipients). By using these process parameters for all formulations, yield values ranging from 70 to 90% were obtained. Specifically, the process yield was observed to decrease in the case of formulations with a higher amount of PEG 400 (e.g., CCPl4), due to the higher tendency of the particles to aggregate and/or adhere to the spray congealing cooling chamber.

CCPl1 was chosen as a model formulation of emulsion-based MPs, while CCPl2 was selected as an example of microemulsion-based MPs. To understand the effect of increasing amounts of the hydrophilic carrier (PEG 400) on the particle structure, the formulation CCPl4 was additionally selected. Furthermore, DCPl2 was chosen to investigate the influence of a different lipid on the MPs morphology. First, particle size, thermal and chemical properties, external morphology and internal structure of b-MPs were characterized. Then, their potential as vehicles for drug delivery was assessed by in vitro release studies.

#### 3.2.1. Size and Surface Morphology of b-MPs

**Figure 4:**
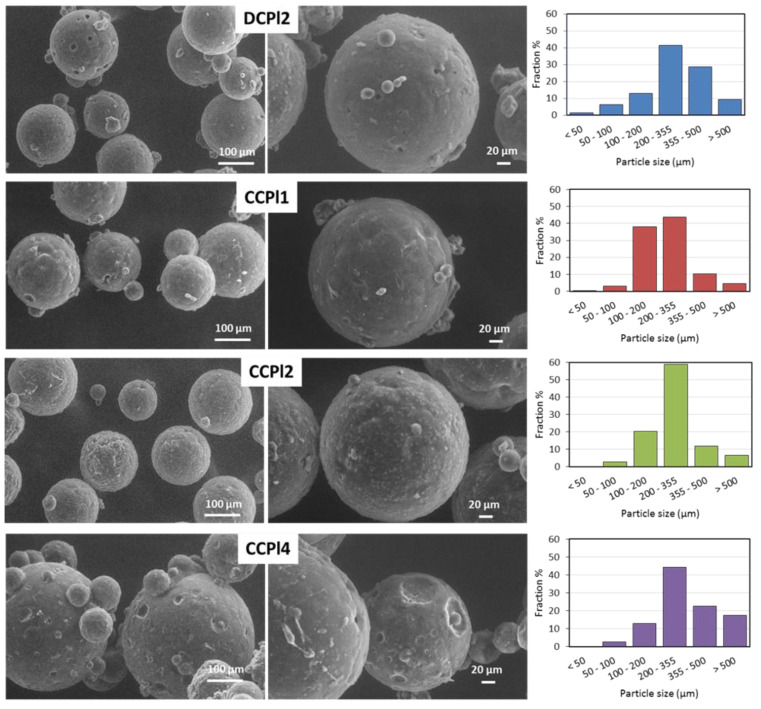
SEM images of DCPl2, CCPL1, CCPl2 and CCPl4 biphasic MPs and their particle size distribution.

SEM and sieve analysis, reported in Figure 4, were employed to gain an understanding of the surface morphology and the size distribution of the biphasic microspheres. SEM images revealed regular and spherical particles with various diameters for all four formulations. Specifically, b-MPs diameters ranged from 50 to 500 µm with the main size fraction between 200 and 355 µm.

The analysis of the particle external morphology showed interesting details. DCPl2 MPs presented many small pores throughout a smooth surface. Those surface pores were probably formed during the MPs cooling where the internal hydrophilic phase (liquid PEG 400) was encapsulated within the solid hydrophobic external phase. In this step, the droplets that were not fully encapsulated and were on (or near) the particle surface were likely to produce the surface pores, as their dimensions are consistent with the size of the droplets observed by optical microscopy for DCPl2 emulsion (Figure 2B). The surface of CCPl1 and CCPl2 MPs were similar with homogeneous regular morphology and absence of pores. Differently, the surface of CCPl4 was characterized by large spherical hollows, probably due to the high amount of PEG 400, leading to the formation of these cavities on the particle surface during the fabrication process.

#### 3.2.2. Thermal and Chemical Properties of b-MPs

DSC and FT-IR spectroscopy were used to provide composition-related information (e.g., molecular structures, solid-state properties, and interactions between components) that can help to understand how the materials are arranged in the b-MPs [32]. Moreover, monitoring the thermal profiles of b-MPs can give important information on the solid-state of the particles (e.g., occurring of polymorphic transformations).

**Figure 5:**
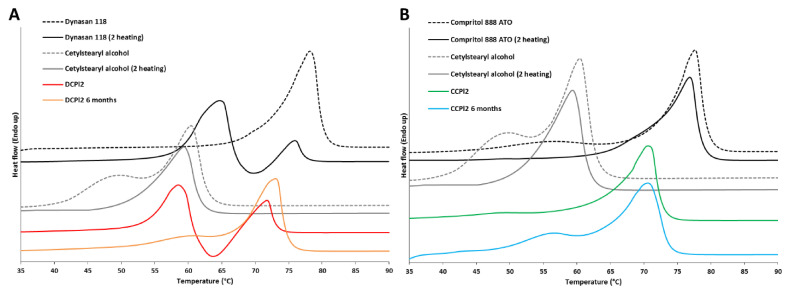
Thermograms of DCPl2 b-MPs immediately after production and after 6 months storage compared to the respective solid raw excipients (Dynasan 118 and cetylsearyl alcohol) subjected to two consecutive heating scans (**A**). Thermograms of CCPl2 b-MPs immediately after production and after 6 months storage compared to the respective solid raw excipients (Compritol 888 ATO and cetylsearyl alcohol) subjected to two consecutive heating scans (**B**).

The thermal behavior of b-MPs is illustrated in Figure 5. It is important to underline that the thermogram of b-MPs, recorded immediately after production, showed the thermal events of formulations obtained by cooling of melted material, and thus, after having been subjected to a heating process. Therefore, to enable a correct comparison between b-MPs and solid excipients, both the thermal profiles of the thermodynamically stable forms (first heating, dashed lines) and of the form solidified after melting (second heating, solid lines) of the excipients were reported. The thermal profile of DCPl2 (Figure 5A) was characterized by three unresolved events: one endothermic peak at 59 °C, one exothermic event at 64 °C and a second endothermic peak at 72 °C. This profile was similar to those obtained from the second heating of the main lipid component, Dynasan 118, which showed an exothermic event (69 °C) placed between two endothermic peaks (65 and 76 °C). This behavior, typical of triglycerides, is related to the melting of the metastable α-polymorph, its solidification into the stable polymorph during the scan, followed by melting of the stable β-form [33]. Rapid crystallization has been reported to promote crystallization in the α-form [34]. Interestingly, a melting peak related to the lipophilic surfactant (cetylstearyl alcohol) was not detected in the thermogram of DCPl2, whereas the melting endotherm of Dynasan 118 was moved to lower temperatures, indicating complete miscibility of lipid and surfactant in the lipophilic phase. In the case of Compritol^®^ 888 ATO (Figure 5B), the main melting endotherm at 77 °C is similar for both raw material and melted-cooled material. Additionally, a weak endothermic event occurring at 45–55 °C is related to the transformation of the pseudohexagonal subcell arrangement of hydrocarbon chains, namely sub-α, into true hexagonal (α) subcell [35,36]. This sub α→α transition observed in the first heating was no longer detected during the second heating, while a shoulder at 65–69 °C was observed, representing the melting of a second α crystalline phase, according to the literature [36,37]. Thus, compared to Dynasan 118, the formation of metastable crystalline forms of Compritol^®^ 888 ATO, consisting of a mixture of mono-, di-, and triglycerides, is more complex as a consequence of the diversity of possible crystalline phases formed upon cooling [35,36,37]. The thermal profile of CCPl2 (Figure 5B) showed a single endotherm at 71 °C, suggesting again the formation of a homogeneous hydrophobic phase with a melting point between the lipid and the surfactant respective melting temperatures.

To investigate the thermal stability of the b-MPs, DSC was performed after 6 months from production. In the case of DPCl2, after 6 months of storage at room temperature, the main thermal event detected was related to the melting of the β-polymorph, indicating that the conversion into the stable crystalline form. The conversion of the α-form of Dynasan 118-based MPs to the stable one is reported to be extremely slow and to take several months [33]. The presence of cetylstearyl alcohol, PEG and Cremophor EL might have accelerated the polymorphic transformation, as previously observed in other studies focused on the addition of liquid lipids [33] and some solid emulsifiers [38] to tristearin. In the case of CCPl1, the main endotherm at about 70 °C did not show significant differences after 6 months of storage. Moreover, a weak endothermic event between 50 and 65 °C was observed in both formulations (DCPl2 and CCPl1). As both lipids exhibited endothermic events close to this temperature range, these transitions might be due to the melting of the α-form of Dynasan and to the sub α→α transition of Compritol, respectively. Alternatively, this endotherm might be related to the melting of the other solid component, cetylstearyl alcohol, which might be subjected to a progressive migration and partial segregation from the main lipid over time.

The FT-IR analysis of DCPl2 and CCPl2 (Figure S3) showed all the main bands of the lipid and the hydrophobic surfactants at unmodified wavenumbers, indicating the absence of modifications of functional groups or strong interactions between the b-MPs components. However, it should be noted the appearance of new bands in the b-MPs spectra (marked with red arrows) at 1349–1352 cm^−1^, 948–950 cm^−1^, 835–840 cm^−1^ and a broad band between 1050–1100 cm^−1^. These bands, which could not be attributed to neither component of the lipophilic phase, were found to correspond to the main signals of PEG, thus indicating the presence of the hydrophilic phase in the b-MPs. Therefore, FT-IR analysis confirmed the successful production of b-MPs with two different phases.

#### 3.2.3. Internal Morphology of b-MPs

The spatial distribution of the hydrophilic and lipophilic phases into the four formulations of b-MPs was determined employing different techniques: CLSM, cross-sectional SEM and Raman mapping.

**Figure 6:**
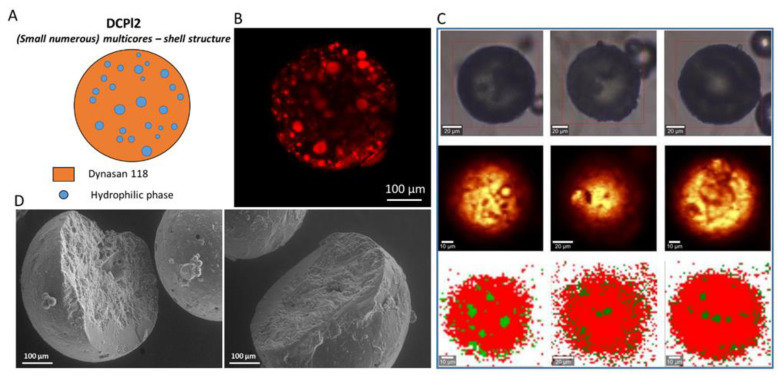
Internal morphology of DCPl2 b-MPs. Schematic illustration of particle structure (**A**). CLSM images, Rho (red color) solubilized in the hydrophilic phase was used as fluorescent probe (**B**). Raman mapping images. The first row (at the top) shows visible pictures; the second row shows Raman images obtained based on the integration of characteristic Raman bands at 2852 and 2886 cm^−1^; the third row shows Raman images: lipophilic phase (red area) and hydrophilic phase (green area) (**C**). SEM images of cross-sections of DCPl2 b-MPs (**D**).

**Figure 7:**
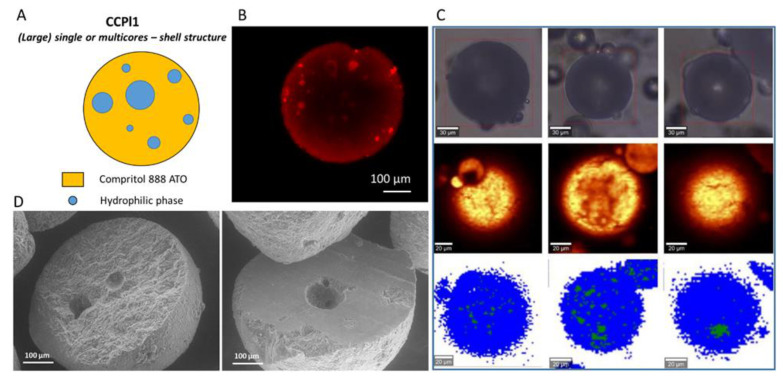
Internal morphology of CCPl1 b-MPs. Schematic illustration of particle structure (**A**). CLSM images, Rho (red color) solubilized in the hydrophilic phase was used as a fluorescent probe (**B**). Raman mapping images. The first row (at the top) shows visible pictures; the second row shows Raman images obtained based on the integration of characteristic Raman bands at 2852 and 2886 cm^−1^; the third row shows Raman images: lipophilic phase (blue area) and hydrophilic phase (green area) (**C**). SEM images of cross-sections of CCPl1 b-MPs (**D**).

**Figure 8:**
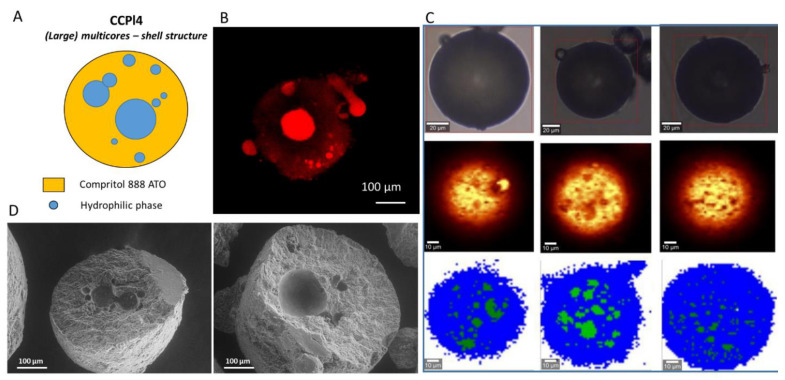
Internal morphology of CCPl4 b-MPs. Schematic illustration of particle structure (**A**). CLSM images, Rho (red color) solubilized in the hydrophilic phase was used as a fluorescent probe (**B**). Raman mapping images. The first row (at the top) shows visible pictures; the second row shows Raman images obtained based on the integration of characteristic Raman bands at 2852 and 2886 cm^−1^; the third row shows Raman images: lipophilic phase (blue area) and hydrophilic phase (green area) (**C**). SEM images of cross-sections of CCPl4 b-MPs (**D**).

**Figure 9:**
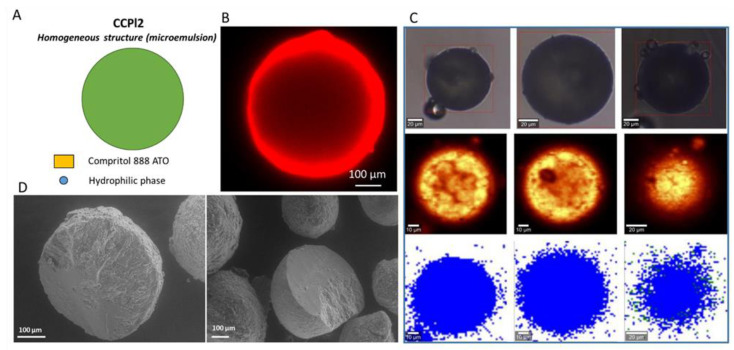
Internal morphology of CCPl2 b-MPs. Schematic illustration of particle structure (**A**). CLSM images, Rho (red color) solubilized in the hydrophilic phase was used as a fluorescent probe (**B**). Raman mapping images. The first row (at the top) shows visible pictures; the second row shows Raman images obtained based on the integration of characteristic Raman bands at 2852 and 2886 cm^−1^; the third row shows Raman images: lipophilic phase (blue area) and hydrophilic phase (green area) (**C**). SEM images of cross-sections of CCPl2 b-MPs (**D**).

CLSM has been selected as a promising technique to investigate b-MPs morphology. It can be applied as a non-destructive method to visualize their internal structure, provided that the carrier matrixes are opportunely labeled with a fluorescent probe [39]. In addition, SEM analysis of cross-sections of b-MPs can provide valuable details on the morphology and appearance of the interior of the particles. Raman mapping is a highly sensitive and precise characterization method based on the detection of Raman scattering diversity of the different materials. Raman microscopy couples a Raman spectrometer to a confocal optical microscope, allowing high magnification visualization of a sample and Raman analysis with a microscopic laser spot [40,41,42]. This technique allowed to accurately determine the spatial distribution of the hydrophilic and lipophilic phase in the b-MPs without the addition of probe molecules, which might represent a potential factor of interference. First, Raman spectra were registered for the raw materials (Figure S4). The registered Raman spectra of Dynasan^®^118, Compritol 888^®^ATO and cetylstearyl alcohol were very similar and it was, therefore, impossible to indicate significant marker bands in order to determine their distribution in the lipophilic phase. Nevertheless, significant differences in the location of key marker bands were observed for hydrophilic ingredients (PEG 400 and Cremophor^®^EL). PEG 400 can be identified by the signals at wavenumbers of 2889, 1477, 1291, 1141, 841 cm^−1^, whereas Cremophor^®^EL indicates key bands at wavenumbers of 2891, 1659, 1446, 1302, 1143 cm^−1^. In Figure S5, Raman spectra for the four b-MPs (DCPI2, CCPI1, CCPI2, CCPl4) are depicted, showing the main bands of the MPs materials. Then, based on spectra profiles of raw materials, Raman maps were generated for the MPs samples. Raman maps obtained based on the integration of characteristic Raman bands (2852 and 2886 cm^−1^) did not allow for the investigation of the spatial distribution of hydrophilic and lipophilic phases in the samples. Therefore, chemometrics was used to analyze Raman mapping data. The application of k-means cluster analysis in the spectral range from 770 to 980 cm^−1^ allowed for a clear differentiation of hydrophilic and lipophilic phases in the b-MPs. This wavenumber range was chosen because it covers the most significant differences in spectral profiles of hydrophilic and lipophilic ingredients (Figure S5). Two hydrophilic ingredients (PEG 400 and Cremophor^®^EL) show a marker band in this wavenumber range, whereas lipophilic components do not show any bands in this spectral range. For each formulation, three individual particles were portrayed and the maps are shown in Figure 6C, Figure 7C, Figure 8C and Figure 9C. The results clearly demonstrate the ability of Raman mapping to discriminate the hydrophilic and lipophilic materials and their distribution within the MPs volume.

Figure 6 shows the internal structure of DCPl2 b-MPs. CLSM analysis (Figure 6B) showed that the fluorescent probe, concentrated in numerous small spherical cores, was distributed within the entire particle volume. According to Raman mapping analysis (Figure 6C), all MPs examined presented multiple hydrophilic cores (green color) embedded in the lipid phase (red color). Numerous small hydrophilic droplets were observed also near the particle surface, while slightly larger hydrophilic cores were observed in the internal space. The internal structure of DCPl2 MPs obtained by Raman mapping analysis was consistent with those obtained by confocal microscopy. Therefore, these data confirmed the suitability of Rho as a fluorescent probe for the inner phase due to its preferential localization in the hydrophilic phase. The cross-sectional SEM image of b-MPs (Figure 6D) revealed a solid matrix characterized by small spherical pores of about 10–15 µm, in line with the CLSM and Raman mapping results. Overall, the internal morphology of DCPl2 b-MPs reflected the structure of the corresponding starting emulsion, which was characterized by numerous well-defined spherical droplets (Figure 2F). DCPl2 b-MPs can be thus described as “multicores-shell MPs” consisting of numerous spherical hydrophilic cores distributed in a crystalline lipid phase.

The analysis of the internal morphology of formulations based on Compritol 888 ATO as the main lipid are shown in Figure 7, Figure 8 and Figure 9 for CCPl1, CCPl4 and CCPl2, respectively. The results revealed the occurrence of different MPs architectures.

The b-MPs CCPl1 (Figure 7) and CCPl4 (Figure 8) exhibited core–shell structures. The CLSM analysis revealed the presence of fluorescently-labeled cores surrounded by the nonfluorescent lipid phase. Thus, the absence of co-surfactant in the b-MPs composition determined the presence of internal cores. The structures depicted by Raman mapping were in line with the internal morphology shown by confocal microscopy. From both the CLSM and Raman results, it appeared that the number and the size of hydrophilic cores increased in CCPl4 b-MPs, as a result of the increased amount of hydrophilic phase (e.g., CCPl4 having the highest PEG 400 amount). A cross-sectional view of CCPl1 and CCPl4 clearly revealed a core–shell configuration, with empty cavities left by the liquid hydrophilic phase. Each CCPl1 b-MPs showed a few round-shaped hydrophilic cores with diameters of ~ 30–100 µm. CCPl4 b-MPs also presented multiple spherical hydrophilic cores of variable size ranging from 30 to 150 µm. With respect to CCPl1, the SEM images of CCPl4 b-MPs showed that a higher particle volume was occupied by the hydrophilic cores, in accordance with the CLSM and Raman imaging results.

The analysis of CCPl2 (Figure 9) revealed a uniform distribution of the fluorescent probe without the appearance of cores. The effect of decreased signal intensity in the center of the MPs was due to the difficulty of the laser to penetrate deeper into the particle matrix. This occurred in all samples but was more evident for particles of large diameters (e.g., CCPl2 in Figure 9B). Whereas CCPl1 and CCPl4 presented multiple cores of PEG 400, CCPl2 did not show any area of fully-separated hydrophilic phase. Apparently, for this formulation, which originated from a microemulsion, the hydrophilic phase was intimately mixed with the hydrophobic phase resulting in extremely small hydrophilic droplets evenly distributed throughout the particle-matrix, so that no appreciable cores were detected. By comparing CCPl1 and CCPl2, it should be noted that the presence of 5% *w/w* of co-surfactant (Cremophor EL) was sufficient to substantially change the internal morphology of the MPs, which passed from a core-shell structure to a uniform system, as a result of the different structure of the starting emulsion. Accordingly, SEM images showed a solid matrix with no trace of hydrophilic cores. Overall, the internal morphology of CCPl2 b-MPs showed a homogeneous structure with an undistinguished distribution of the two phases, consistently with the structure of the starting microemulsion.

#### 3.2.4. Drug Release from b-MPs

Rho, as a water-soluble dye, was also used to evaluate the dissolution behavior of b-MPs with different microstructures. The determination of Rho content in b-MPs of the selected particle size fraction (250–355 µm) was consistent with the amount of Rho added to the formulation as the encapsulation efficiency was always high. In fact, these values varied from 91.0% to 98.3% for the various formulations and the mean value was 95.5 ± 2.6%. Such a value is considerably higher compared to solvent-based emulsion methods and similar to those obtained with spray congealing process using one-phase materials [22,43,44].

**Figure 10:**
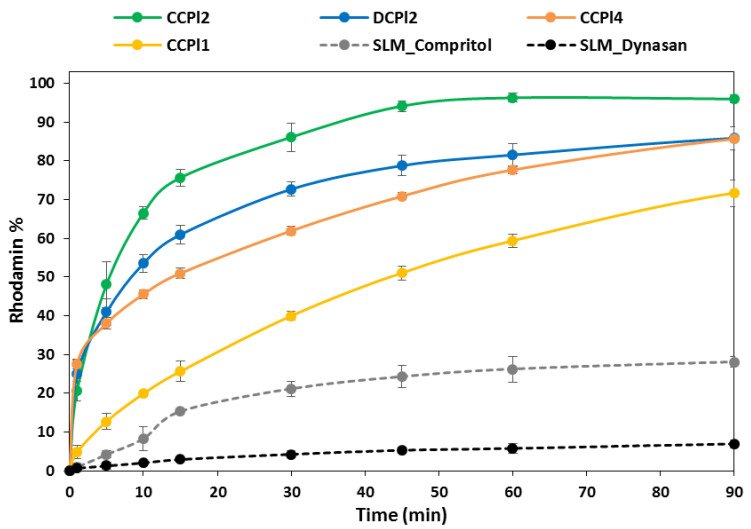
Release profiles of Rho from b-MPs compared with traditional solid lipid microparticles (SLMs) with simple lipid matrix (Compritol or Dynasan). The particle size was 250–355 µm for all samples.

Rho release profiles from different formulations of b-MPs having PEG 400 as the inner phase is reported in Figure 10. The release profiles of all b-MPs were enhanced with respect to matrix systems (Compritol or Dynasan SLMs). Specifically, CCPl2 determined the fastest release: about 20% of the total amount of loaded Rho was released within the first minute of the test and 94% of the total amount was released after 45 min. Slightly lower release rates were obtained by replacing Compritol with Dynasan as the main lipid (DCPl2), although a burst release of about 25% was observed in 1 min. Conversely, b-MPs without co-surfactant (CCPl1 and CCPl4) showed a controlled release of the hydrophilic dye: in the case of CCPl1, 72% of Rho was released after 90 min with a very low burst release (about 4.8% in 1 min). It was interesting to note that by increasing the content of PEG 400 from 20% (CCPl1) to 30% (CCPl4), the rate of drug release did not increase, although a high burst effect of about 30% was observed for CCPl4.

Previous studies [45] evidenced that Compritol 888ATO-based microparticles maintained their structural integrity after 120 min of incubation in simple aqueous media (without enzymes) with only minor changes of the surface structure. The drug release in such SLMs was thus controlled by diffusion through the pores created by the depleted drug. Here, the addition of a hydrophobic surfactant (e.g., cetylstearyl alcohol) and the presence of liquid hydrophilic cores in the b-MPs can be responsible for the enhanced dye release with respect to the simple matrix systems. It has been previously observed that the introduction of lipid additives either solid (stearic acid, cetyl alcohol, or cetyl esters) [46] or liquid [33] lead to increased drug release from spray congealed microparticles. Moreover, results showed that the introduction of small amounts (5–10% *w/w*) of Cremophor EL in the hydrophilic phase led to a significant modification of the release profiles. This might be due to the amphiphilic nature of Cremophor EL, which enhanced the wettability of the particle surfaces, favoring the water permeation through the pores created by the hydrophilic cores once mixed with the dissolution bulk, thus favoring the release of the encapsulated drug. Further, as SEM images (Figure 4) evidences several micro-holes on the b-MPs surface, especially for DCPl2 and CCPl4, it can be hypothesized that the water-miscible PEG cores behave like “pore formers” by creating several microchannels through which the dissolved dye can diffuse. Accordingly, in the case of Compritol-based b-MPs, it appeared that a microemulsion-like structure (CCPl2) with extremely small hydrophilic droplets homogeneously distributed through the lipid phase determined a faster release compared to core-shell structures (CCPl1 and CCPl4), where the drug is localized in the internal cores surrounded by a hydrophobic layer.

Additional release studies were performed in order to evaluate the effect of different formulation variables of Compritol-based emulsions, such as different lipid:surfactant ratios and different hydrophilic phases (Figure S6). By increasing the lipid:surfactant ratio from 3:1 to 6:1, only minimal variations were observed (Figure S6A). For example, compared to CCPl2, the drug release was only slightly reduced with the same formulation with Compritol:cetylstearyl alcohol ratio of 6:1. Finally, the effect of different hydrophilic phases on the release behavior of b-MPs was investigated and the results are reported in Figure S6B. By replacing liquid PEG 400 with solid hydrophilic carriers, such as PEG 4000 (CCPs2) or Gelucire 48/16 (CCGe1), similar release profiles were noted. The highest release rate was obtained for MPs with Poloxamer 118 as the hydrophilic phase (CCPo1). Therefore, b-MPs containing surface-active excipients (Gelucire 48/6 and Poloxamer 188), as the inner hydrophilic phase determined immediate drug release, consistently with the starting microemulsion (Figure 2E). It should be noted that the release profile obtained by the system containing solid PEG (CCPs2) was equivalent to that of CCPl2, containing liquid PEG, although the different emulsion structures (Figure 2D,E). It appeared, therefore, that the hydrophilic character of PEG, rather than the emulsion structure, dictated the release behavior.

Overall, it appeared that the most critical feature dictating the release behavior is the amount of co-surfactant Cremophor EL. Secondly, the nature of the main lipid also contributes to determining the release profiles. Moreover, the type and amount of hydrophilic carrier influenced the release, while its physical state (solid or liquid) had minor importance. It was interesting to note that among the different formulations tested, CCPl1 showed a constant and sustained release of the model compound, with minimal bust effect. The release profile of CCPl1 MPs appeared, therefore, very promising, with possible application in the formulation of delivery systems for hydrophilic small or large drug molecules requiring a sustained release to be completed within a few hours, e.g., oral delivery of biologics. On the other hand, the solubilization of the API in the inner phase (PEG cores) could determine an enhanced release, and therefore, be potentially useful for poorly water-soluble drugs. Finally, the release behavior of CCPl2 formulation suggests a possible application as an immediate release dosage form.

## 4. Conclusions

We have successfully developed, for the first time, biphasic microparticles (b-MPs) by employing a solvent-free approach based on spray congealing, which enabled the transformation of non-aqueous melt emulsions into spherical solid particles with good process yield. SEM, CLSM and Raman mapping showed that different microstructures can be obtained. The distribution of the two phases reflected the starting liquid system structure: microemulsions generated particles with a homogeneous structure and undistinguished phase distribution, while coarse emulsions with micron- or submicron-sized dispersed droplets led to the formation of particles with a hydrophilic multicore-lipophilic shell structure. These different architectures strongly influenced the b-MPs release behavior, displaying either an enhanced or a sustained release profile, suggesting the potential of b-MPs as a versatile drug delivery system. Compared to one-phase lipid particles (i.e., SLMs), which allowed very slow drug release with most dye retained into the lipid system, the release performances of b-MPs were more tunable. Notably, the release behavior of b-MPs could be influenced by the core and shell materials and by their relative amounts, in addition to the properties of the specific loaded drug. Indeed, whereas the choice of a specific lipid carrier or a combination of different lipids may be used to modify the release behavior from SLMs, the presence of a biphasic structure with two compartments allowed a broader formulation space and a higher modified release potential.

It will be interesting in the near future to study the behavior of these systems as drug delivery platforms, focusing on the selection of suitable lipid excipients and additives according to specific release targets and stability issues. Indeed, the addition of an API with varying loading amount, location (either in the inner or outer phase of the particle) and drug type could affect both the structure of the b-MPs and the release behavior. Moreover, the lipid digestion carried out by lipases in the gastrointestinal tract should be considered as it could potentially impact drug release performances from b-MPs.

Overall, the strategy proposed in this study provides a feasible and cost-effective way of fabricating biphasic particles with composition-dependent internal structure and tunable release behavior, with promising application in the pharmaceutical, nutraceutical and food industries.

## Figures and Tables

**Table 1:** Characteristics of the materials used for the production of biphasic MPs.

Material	Chemical Composition	T_m_ (°C)	HLB
**Lipid**			
Dynasan^®^118	C18 triglycerides	72–73	-
Compritol^®^888 ATO	C16-22 mono-, di- and tri-glycerides	69–72	2
**Hydrophobic surfactant**			
Cetylstearyl alcohol	Cetyl and stearyl alcohol	50–60	4.7
Span^®^60	Sorbitan monostearate	56–58	4.7
**Hydrophilic carrier**			
PEG 400	Polyethylene glycol (MW 400)	-	-
PEG 4000	Polyethylene glycol (MW 4000)	66–68	-
Poloxamer 188	Block copolymers of poly(ethylene oxide) and poly(propylene oxide)	58–60	>24
Gelucire^®^48/16	Polyethylene glycol (MW 1500) esters of C16-18 fatty acids	46–50	12
**Liquid hydrophilic co-surfactant**			
Cremophor^®^EL	Polyoxyl 35 Hydrogenated Castor Oil	-	12–14

**Table 2:** Composition of the emulsions with Dynasan^®^118 as lipid.

Sample	Lipid-Main Surfactant % (*w/w*) (3:1)		Hydrophilic Carrier % (*w/w*)	Co-Surfactant % (*w/w*)
	Dynasan^®^118- Cetylstearyl alcohol	Dynasan^®^118- Span 60	PEG 400	Cremophor^®^EL
DCPl2	80	-	15	5
DSPl2	-	80	15	5

**Table 3:** Composition of the emulsions with Compritol^®^888 ATO as lipid.

Sample	Lipid-Main Surfactant % (*w/w*) (3:1)		Hydrophilic Carrier % (*w/w*)				Co-Surfactant % (*w/w*)
	Compritol^®^- Cetylstearyl alcohol	Compritol^®^- Span 60	PEG 400	PEG 4000	Poloxamer^®^ 188	Gelucire^®^ 48/16	Cremophor EL
CCPl1	80	-	20	-	-	-	-
CCPl2	80	-	15	-	-	-	5
CCPl3	75	-	15	-	-	-	10
CCPl4	70	-	30	-	-	-	-
CCPl2 *	80	-	15	-	-	-	5
CCPl3 *	75	-	15	-	-	-	10
CSPl2	-	80	15	-	-	-	5
CCPs2	80	-	-	15	-	-	5
CCPo1	80	-	-	-	20	-	-
CCGe1	80	-	-	-	-	20	-

* The lipid:main surfactant ratio is 6:1 instead of 3:1.

## Supplementary Materials

The following are available online at https://www.mdpi.com/article/10.3390/pharmaceutics14010054/s1, Figure S1: Excitation and emission fluorescence spectra of Rho, Figure S2: Calibration curve of Rho using fluorescence measurements, Figure S3: FT-IR spectra of DCPl2 and CCPl2 b-MPs compared to the respective solid raw excipients, Figure S4: Raman spectra of the raw materials, Figure S5: Raman spectra of the b-MPs, Figure S6: Drug release profiles of Compritol-based b-MPs with different lipid:surfactant ratio and various hydrophilic phases.
