# Supplementary material for: Solvent-Free Fabrication of Biphasic Lipid-Based Microparticles with Tunable Structure
Source: Pharmaceutics. 2021 Dec 27;14(1):54. doi: 10.3390/pharmaceutics14010054 (PMC8780016; doi:10.3390/pharmaceutics14010054)
Supplement: Supplementary file 1 [file pharmaceutics-14-00054-s001.zip › pharmaceutics-1498484-supplementary.pdf]

pharmaceutics

MDPI

Supplementary Materials: Solvent-Free Fabrication of Biphasic Lipid-Based Microparticles with Tunable Structure

Serena Bertoni, Beatrice Albertini, Joanna Ronowicz-Pilarczyk, Natalia Calonghi and Nadia Passerini

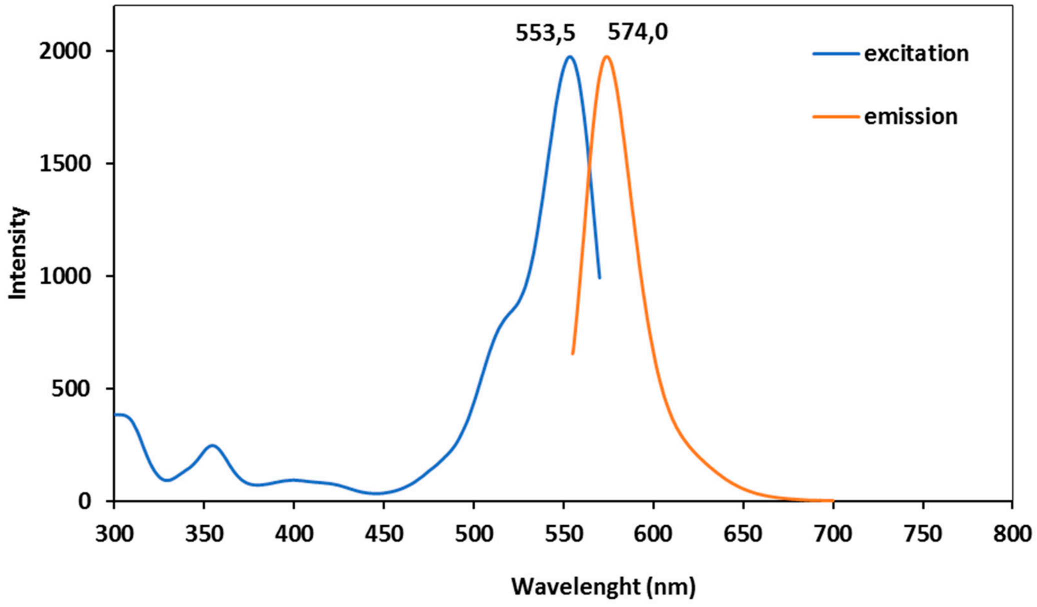

Figure S1. Excitation and emission fluorescence spectra of Rho.

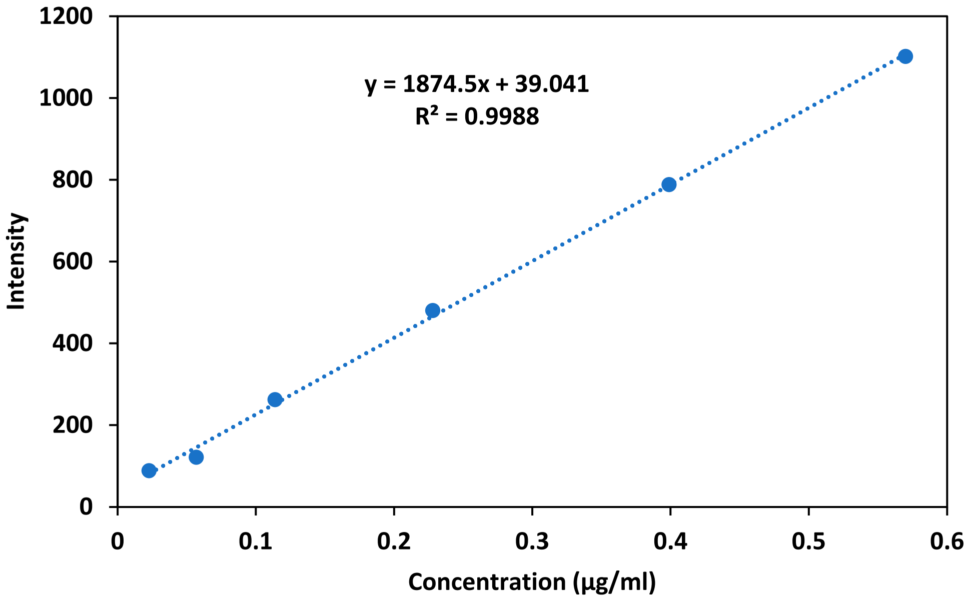

Figure S2. Calibration curve of Rho using fluorescence measurements.

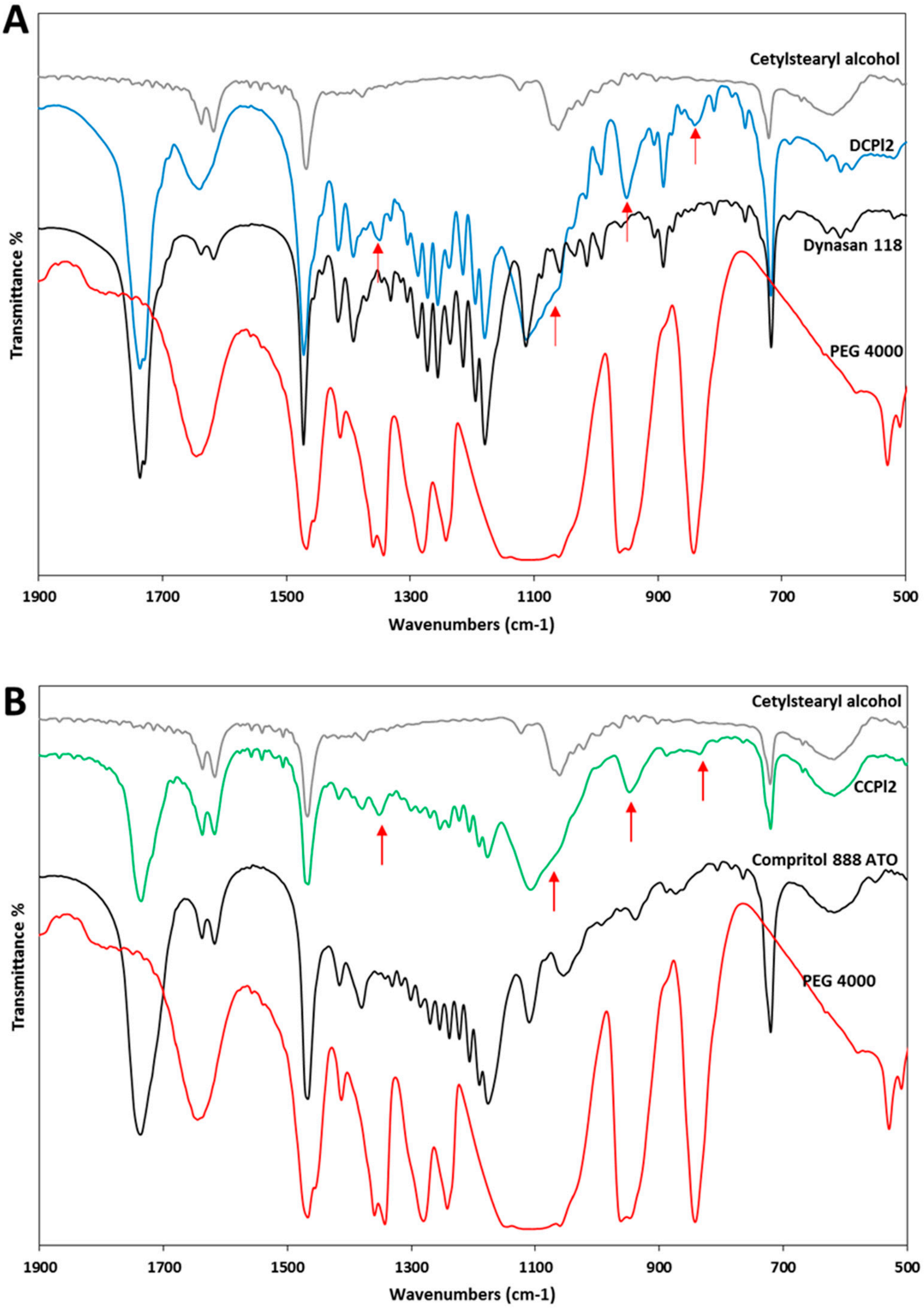

Figure S3. FT-IR spectra of DCPl2 b-MPs compared to the respective solid raw excipients (**A**) and CCPl2 b-MPs compared to the respective solid raw excipients (**B**). Red arrows indicate the bands in the b-MPs spectra attributed to the hydrophilic phase.

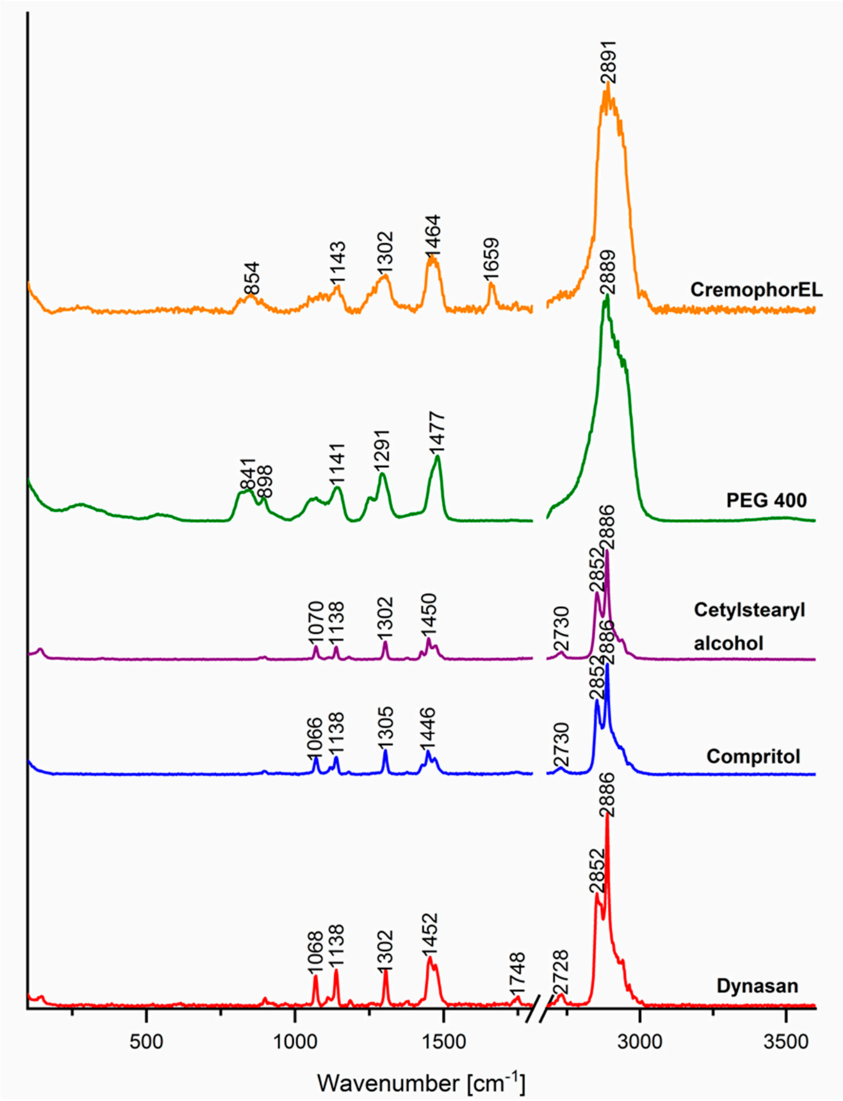

Figure S4. Raman spectra recorded for raw materials: Dynasan®118 (red line), Compritol® 888 ATO (blue line), cetylstearyl alcohol (purple line), PEG 400 (green line) and Cremophor®EL (orange line).

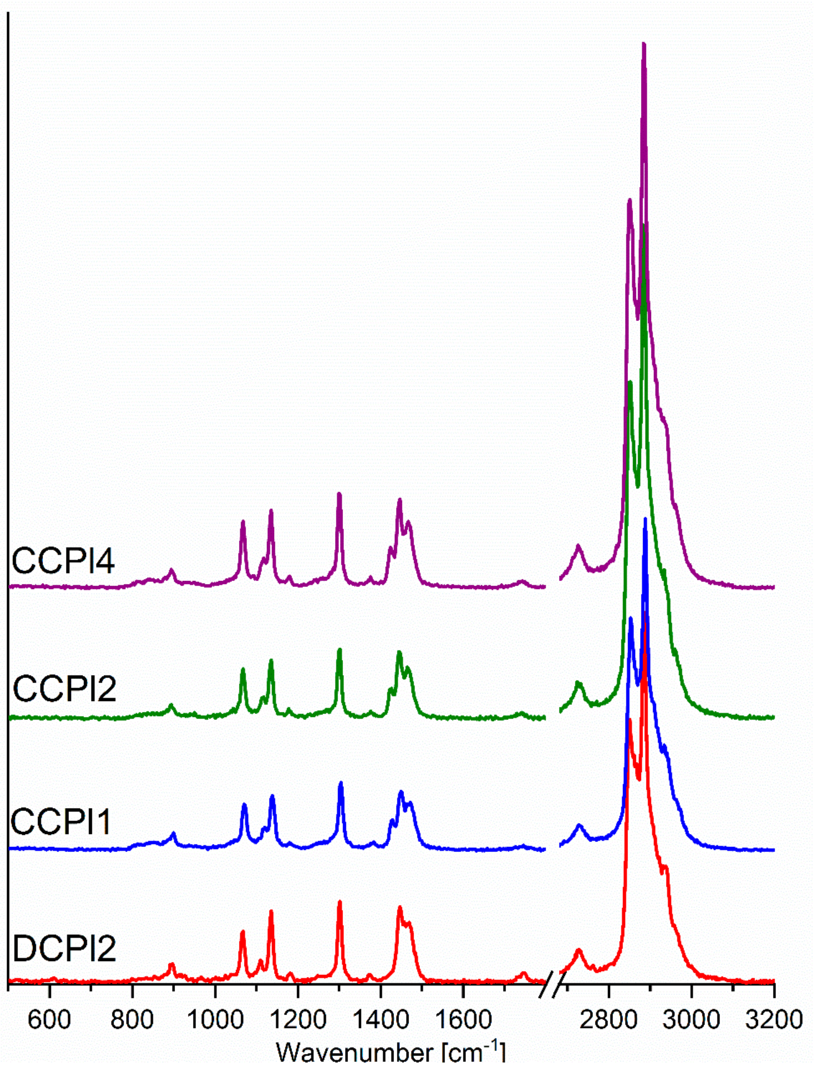

Figure S5. Raman spectra recorded for b-MPs: DCPI2 (red line), CCPI1 (blue line), CCPI2 (green line), CCPI4 (purple line).

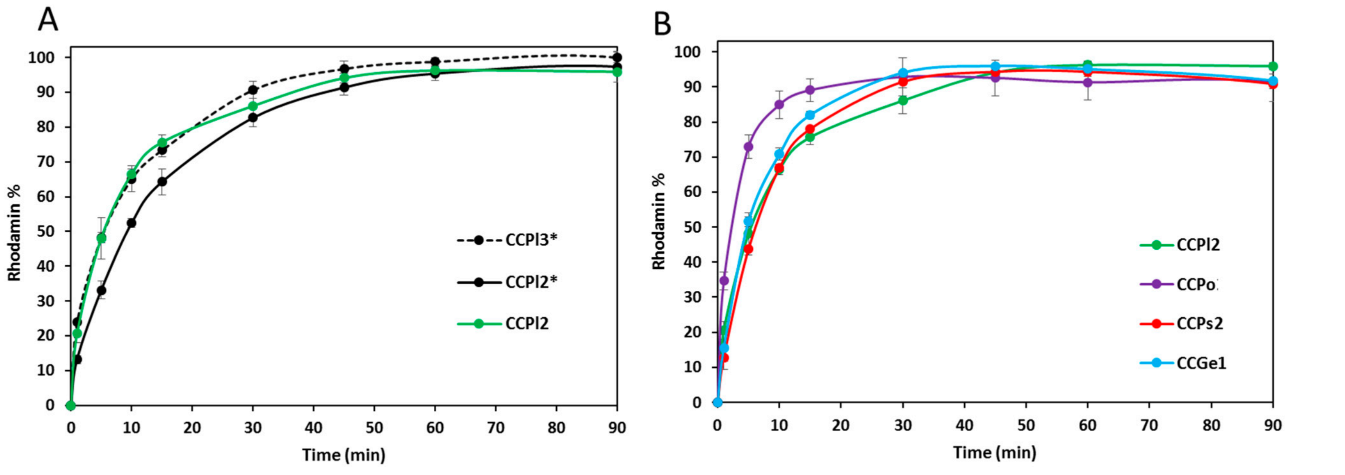

Figure S6. Effect of the lipid:surfactant ratio (**A**); and of various hydrophilic phases (**B**) on drug release from Compritol-based biphasic MPs (250-355 μm).
